# Population pharmacokinetics of imipenem in different populations for individualized dosing: a systematic review

**DOI:** 10.3389/fphar.2025.1738055

**Published:** 2026-01-14

**Authors:** Ping Zhang, Yuhua Zhao, Jianping Zhu, Yi Yang, Gang Liang, Xia Wang, Zhenwei Yu

**Affiliations:** 1 Department of Pharmacy, Sir Run Run Shaw Hospital, School of Medicine, Zhejiang University, Hangzhou, China; 2 Department of Pharmacy, Affiliated Xiaoshan Hospital, Hangzhou Normal University, Hangzhou, China; 3 Department of Pharmacy, The Affiliated Taian City Central Hospital of Qingdao University, Taian, China; 4 Research Center for Clinical Pharmacy, College of Pharmaceutical Science, Zhejiang University, Hangzhou, China

**Keywords:** covariates, creatinine clearance, imipenem, model, population pharmacokinetics

## Abstract

**Background:**

Imipenem is a broad-spectrum carbapenem antibiotic for severe infections with significant pharmacokinetic (PK) variability. This review systematically synthesized published population pharmacokinetic (popPK) studies to identify key covariates and guide individualized dosing for patients with various conditions.

**Methods:**

A systematic PubMed and Web of Science search identified imipenem popPK models. Studies employing nonlinear mixed-effects modeling in patients with various conditions were included, and data were extracted independently by two reviewers via a standardized form. The study characteristics and PK parameter estimates were compared.

**Results:**

This systematic review of 18 popPK studies revealed that imipenem PKs were predominantly characterized by two-compartment models. The clearance of imipenem varied from 4.79 to 16.2 L/h in adults. Creatinine clearance (CLcr) was the most consistent and significant covariate for imipenem clearance, whereas body weight (BW) was frequently identified for volume of distribution. Other clinically relevant covariates, including the glomerular filtration rate (GFR), age, and serum ALB level, were also incorporated into the final models for specific patient subpopulations. All models applied internal validations, such as bootstrap and visual predicative check, but only three studies performed external validation.

**Conclusion:**

This review systematically integrates existing popPK models of imipenem, highlighting renal function and BW as key covariates. This study provides valuable insights for individualized dosing while identifying critical research gaps, particularly the need for external validation and focused studies in special populations.

## 1 Introduction

Imipenem, a broad-spectrum carbapenem antibiotic, possesses potent antibacterial activity against a diverse range of gram-positive and gram-negative bacteria, as well as anaerobic organisms (Dingle and Pitout, 2022). Its bactericidal action is mediated through the inhibition of bacterial cell wall synthesis (O’Donnell and Lodise, 2022). In the clinic, imipenem is widely utilized for the management of severe bacterial infections, including sepsis, pneumonia, intra-abdominal infections, and urinary tract infections (Chang et al., 2023; Fratoni et al., 2022; Heo, 2021; Marino et al., 2025; Titov et al., 2021). The primary objective of its dosing regimen is to maximize the duration during which plasma concentrations exceed the pathogen’s minimum inhibitory concentration (MIC) (Song et al., 2025). Consequently, imipenem is commonly coadministered with cilastatin, a renal dehydropeptidase inhibitor, to prevent renal metabolism and inactivation, thereby ensuring that a sufficient amount of intact drug reaches the target site and maintains excellent clinical efficacy (Zhanel et al., 2018).

Despite its strong antibacterial potency, the clinical use of imipenem is accompanied by a range of noteworthy adverse effects. An analysis of 2,574 adverse event reports related to imipenem revealed that over half of the events involved individuals aged over 60 years. Previously unreported adverse reactions, including brain atrophy and delirium, have also been identified (Jia et al., 2025). Among the most concerning adverse effects is dose-dependent central neurotoxicity (Sutter et al., 2015). Reported potential targets for carbapenem-induced neurotoxicity include the GABA_A_ receptor, glutathione S-transferase Pi, glutathione S-transferase Mu 1, and glutathione S-transferase A2 (de Lima et al., 2025). The incidence of this complication is significantly elevated in patients receiving high doses, those with renal impairment leading to drug accumulation, and individuals with preexisting central nervous system disorders (Miller et al., 2011). Furthermore, similar to other β-lactam antibiotics, imipenem may induce gastrointestinal disturbances, allergic manifestations such as skin rashes, and occasional eosinophilia (Foong et al., 2016; Hamao et al., 2025; Sivanandy et al., 2024). Notably, although cilastatin is employed to inhibit renal organic anion transporters (OATs) and prevent imipenem-induced nephrotoxicity, imipenem/cilastatin administration has been associated with alkaline urine, polyuria, crystalluria, and elevated plasma levels of urea, creatinine, and uric acid, indicating that potential nephrotoxic risk still requires close monitoring (Huo et al., 2019; Tahri et al., 2018). A more serious long-term challenge is the emergence and dissemination of carbapenem-resistant strains driven by inappropriate and extensive use, particularly when drug exposure remains at subtherapeutic levels for prolonged periods, exerting substantial selective pressure and posing a significant threat to global public health (Lechtig-Wasserman et al., 2021).

Given the complexities associated with its clinical application, the pharmacokinetic (PK) profile of imipenem is highly important. This antibiotic exhibits time-dependent bactericidal activity, the efficacy of which is commonly quantified by %*f*T_>MIC_, the percentage of the dosing interval during which plasma concentrations exceed the MIC (Fratoni et al., 2023; Khan et al., 2025). Accordingly, a precise understanding of its PK behavior and the maintenance of adequate drug exposure duration are essential for optimizing therapeutic outcomes and preventing the emergence of resistance. In clinical practice, significant interindividual and intraindividual variability in imipenem PK has been observed (Zou et al., 2019). This variability may be influenced by multiple factors, including patient age, body weight (BW), renal function, comorbid conditions, and drug‒drug interactions. For example, impaired renal function can lead to reduced drug clearance and subsequent drug accumulation (Bricheux et al., 2018). However, in patients with obesity, the volume of drug distribution tends to increase, potentially necessitating adjustments to the dosing regimen to maintain effective plasma drug concentrations (Chen et al., 2024). Furthermore, variations in drug concentrations across different infection sites can impact treatment efficacy (Alikhani et al., 2025). Such PK variability presents substantial challenges for appropriate clinical use, highlighting the urgent need to develop individualized dosing strategies that ensure both efficacy and safety.

To address these challenges, population pharmacokinetic (popPK) modeling offers a valuable methodological approach. PopPK utilizes sparsely collected data from routine clinical settings and applies nonlinear mixed-effects models to simultaneously estimate typical population parameter values (fixed effects) as well as interindividual and residual variability (random effects) (Yao et al., 2025). This enables a deeper understanding of the key factors driving PK differences within specific patient subgroups. Although many studies have been conducted to develop popPK models for imipenem, these investigations are dispersed across diverse patient populations. The final model structures and the significant covariates identified often vary, and a systematic integration and comparison are currently lacking. Therefore, this review aims to comprehensively synthesize and systematically evaluate published popPK studies on imipenem. It focuses on assessing and analyzing the structural model employed, the key covariates identified along with their quantitative influences, and comparing the application and validation of these models across various special populations.

## 2 Methods

### 2.1 Search of published population pharmacokinetic models

A systematic literature search was performed via PubMed and Web of Science to identify published popPK models of imipenem. The search covered the period from database inception until August 2025. The following key terms were used: “imipenem” AND (“population pharmacokinetics” OR “population pharmacokinetic” OR “pharmacokinetic analysis” OR “pharmacokinetic model” OR “NONMEM” OR “nonlinear mixed effect model”). All the retrieved articles were thoroughly reviewed and cross-verified by two independent investigators.

### 2.2 Inclusion and exclusion criteria

Studies were included if they met the following criteria: (1) involved human subjects receiving imipenem therapy (healthy volunteers or patients); (2) employed nonlinear mixed-effects modeling for pharmacokinetic analysis; (3) provided a complete popPK model description; and (4) were published in English.

The exclusion criteria were as follows: (1) studies using noncompartmental or nonparametric methods; (2) non-research study or secondary publications; (3) insufficient details on model structure or parameter estimates; (4) studies limited to external validation of existing models; (5) applications of previously published models without new modeling efforts; and (6) studies focused on combinations other than imipenem-cilastatin, such as imipenem-cilastatin-relebactam.

### 2.3 Data extraction

Data were extracted independently by two reviewers via a predesigned standardized form. Any discrepancies were resolved through discussion or by a third reviewer. The extracted information included the following: (1) study design features: year of publication, country, study type (prospective/retrospective), population description, sample size, dosing regimen, sampling schedule, bioanalytical methodology, etc.; (2) participant demographics: age, sex, BW, etc.; and (3) popPK model characteristics: compartmental model, software used, model evaluation techniques, parameter estimates, and significant covariates, etc.

## 3 Results

### 3.1 Literature search and study inclusion

**FIGURE 1:**
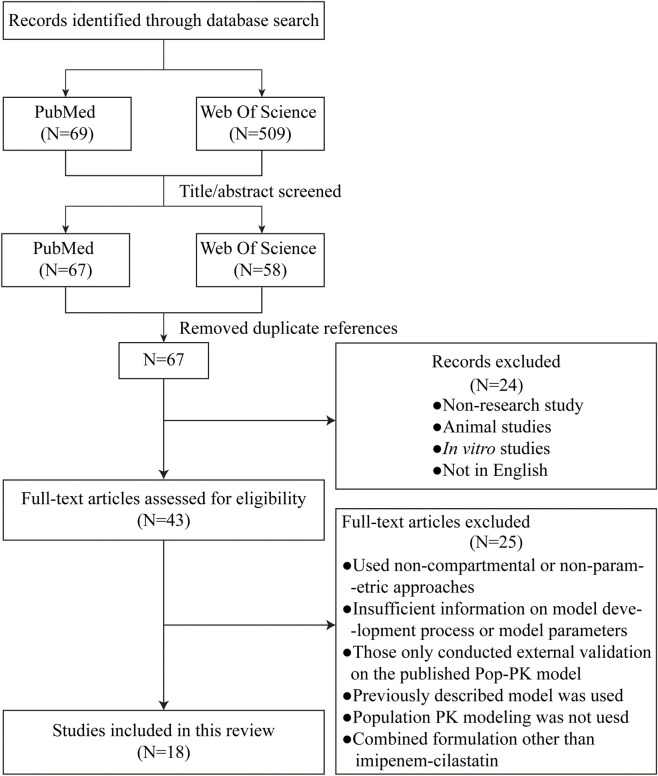
Flow chart of the article retrieval and screening process.

A total of 69 (PubMed) and 509 (Web of Science) potentially relevant articles were initially identified through the implemented search strategies. After screening the titles and abstracts, 67 (PubMed) and 58 (Web of Science) articles remained for further evaluation. After removing duplicates and performing a full-text assessment, 18 studies meeting the inclusion criteria were ultimately included in this review (Bai et al., 2024; Chen et al., 2020b; Couffignal et al., 2014; Dao et al., 2022; de Velde et al., 2020; Dinh et al., 2022; Dong et al., 2019; Ikawa et al., 2008; Jaruratanasirikul et al., 2021; Lafaurie et al., 2023; Lamoth et al., 2009; Li and Xie, 2019; Li et al., 2020; Nguyen et al., 2021; Por et al., 2021; Truong et al., 2025; Wang et al., 2024; Yoshizawa et al., 2013) (Figure 1). The key characteristics of these selected studies are summarized in Table 1. The included patients were from 10 different countries. Five studies were conducted in China, two in France, two in Japan, two in Switzerland, two in the Netherlands, and two in Vietnam. In the view of study design, 10 studies are prospective and the other 8 are retrospective. Single studies were reported from Belgium, Thailand, and the United States. The publication years of these articles spanned from 2008 to 2025. Considerable variation in sample size was observed across the studies, ranging from 10 patients (Ikawa et al., 2008) to 247 patients (Chen et al., 2020b), but the sample sizes for most studies are limited. The study populations were predominantly composed of critically ill patients; however, several studies have focused on specific groups, such as patients with burns, abdominal infections, hematological malignancies, or neutropenia. Patients with organ support, such as CRRT and ECMO, were also analyzed. Additionally, the target populations were not limited to adults but also included neonates and children.

**TABLE 1:** Demographics of patients for all population pharmacokinetic studies included in this review.

Study	Author and year	Country	Study type	Patient group	Number of patients	Sample size	Gender (M/F)	Age (year)	Weight (kg)
1	Ikawa et al. (2008)	Japan	Prospective	Patients with intraabdominal infections	10	NR	NR	43.7 ± 14.9	56.7 ± 10.5
2	Lamoth et al. (2009)	Switzerland	Retrospective	Febrile Neutropenic Patients with Hematological Malignancies	57	159	44/13	58 (17–78)	73 (41–135)
3	Yoshizawa et al. (2013)	Japan	Retrospective	Neonates and children	Neonates: 60 Children: 39	Neonates: blood: 335 urinary: 108 Children: blood: 230 urinary: 155	Neonates: 30/30; Children: 26/13	Neonates: 0.0288 ± 0.0227; Children: 9.61 ± 3.16	Neonates: 2.93 ± 0.7 Children: 29.5 ± 10.9
4	Couffignal et al. (2014)	France	Prospective	Critically ill patients with suspected ventilator-associated pneumonia	51	297	41/10	60 (28–84)	77 (45–126)
5	Li and Xie (2019)	Belgium	Retrospective	Critically Ill patients with CRRT	20	134	16/4	55.5 (42.8–69.8)	72 (62.5–82.5)
6	Dong et al. (2019)	China	Prospective	Children with hematological malignancies	56	136	30/26	4.86 ± 2.33	18.65 ± 6.90
7	Li et al. (2020)	China	Prospective	Critically Ill patients with CRRT	30	blood: 209 effluent: 174	23/7	61.67 ± 19.77	70.2 ± 13.68
8	Chen et al. (2020b)	China	Retrospective	Critically Ill patients with/without ECMO	247	580	167/80	67 (20–97)	65 (37.5–110.0)
9	de Velde et al. (2020)	Netherlands	Retrospective	Critically ill patients	26	138	18/8	51 (39–54)	75 (66–85)
10	Nguyen et al. (2021)	Vietnam	Prospective	AECOPD	44	84	41/3	65 (60-72)	50 (47–55)
11	Jaruratanasirikul et al. (2021)	Thailand	Prospective	Critically ill patients with/without ECMO	50	534	35/15	56.2 (40.95–66.6)	62.9 (52.8–70.0)
12	Por et al. (2021)	USA	Prospective	Burn patients with/without CVVH	23	81	6/17	With CVVH: 55 ± 19.99 Without CVVH: 51.09 ± 19.03	With CVVH: 89.6 ± 22.38 Without CVVH: 105.06 ± 28.66
13	Dao et al. (2022)	Switzerland	Retrospective	Neonates	82	173	38/44	GA: 26.9 (24.2–41.3) w PNA: 21 (2.1–153) d PMA: 31 (25.6–48.3) w	1.16 (0.5–4.1)
14	Dinh et al. (2022)	Vietnam	Prospective	Critically-Ill patients	24	139	18/6	57.5 ± 19.9	51.3 ± 8.6
15	Bai et al. (2024)	China	prospective	Critically Ill sepsis	51	196	33/18	56.45 ± 18.76	70.21 ± 72.01
16	Lafaurie et al. (2023)	France	Prospective	Neutropenic adult patients	16	118	7/9	37 (18.3–78.3)	65.5 (48–101)
17	Truong et al. (2025)	Netherlands	Retrospective	Critically ill and non-critically ill patients	151	322	151/0	63 (51–72)	70 (61.2–82)
18	Wang et al. (2024)	China	Retrospective	Elderly patients	120	370	78/42	72 (68, 81)	65 (59, 65.33)

The age and weight values are presented as the means ± SDs or medians (ranges). USA, United States of America; M/F, Male/Female; AECOPD, Acute Exacerbations of Chronic Obstructive Pulmonary Disease; w, week; d, day; CRRT, Continuous Renal Replacement Therapy; CVVH, continuous venous hemofiltration; ECMO, extracorporeal membrane oxygenation; GA, gestational age; PNA, postnatal age; PMA, Postmenstrual age; NR, no record.

As presented in Supplementary Table S1, the recommended therapeutic dose of imipenem for most adult patients with infections is 1,000–2,000 mg per day, which is administered in three to four intravenous infusions. For children and neonates weighing less than 40 kg, a dosage of 15 mg/kg every 6 h is recommended, with a maximum daily dose not exceeding 2 g. In the present review, the majority of the dosing regimens were consistent with these recommendations; however, five studies (Dinh et al., 2022; Jaruratanasirikul et al., 2021; Li and Xie, 2019; Por et al., 2021; Wang et al., 2024) reported a maximum daily dose of 4,000 mg. Furthermore, the imipenem dosage was not specified in Truong et al. (2025) Study. The timing of sample collection varied considerably across the studies. For example, in Wang et al. (2024) Study, only trough concentration samples were collected, whereas in de Velde et al. (2020) Study, blood samples were obtained at peak, intermediate, and trough time points. The remaining studies collected samples at multiple time points. Nearly all studies employed high-performance liquid chromatography with ultraviolet detection (HPLC-UV) for the quantification of imipenem concentrations in blood samples. An exception was Lamoth et al. (2009) Study, which utilized HPLC alone, and Chen et al., (2020b) Study and Dao et al. (2022) Study, in which liquid chromatography‒tandem mass spectrometry (LC‒MS/MS) was applied.

### 3.2 Population pharmacokinetic analyses

PopPK modeling approaches across the included studies are summarized in Table 2. The most frequently employed software was NONMEM, which was applied in 11 investigations to generate popPK models (Chen et al., 2020b; Dao et al., 2022; de Velde et al., 2020; Dong et al., 2019; Ikawa et al., 2008; Jaruratanasirikul et al., 2021; Lamoth et al., 2009; Li and Xie, 2019; Truong et al., 2025; Wang et al., 2024; Yoshizawa et al., 2013). In contrast, Monolix was used in four studies (Couffignal et al., 2014; Dinh et al., 2022; Lafaurie et al., 2023; Nguyen et al., 2021). Alternative tools included Phoenix NLME, which was implemented in two studies, and Pumas, which was utilized in one study (Bai et al., 2024; Li et al., 2020; Por et al., 2021). Model evaluation relied on both basic and advanced internal validation techniques. Most studies had performed internal validation. Goodness-of-fit (GOF) plots are routinely examined, and methods such as bootstrapping and visual predictive checks (VPCs) are also commonly employed to assess model robustness. In contrast, only three studies had performed external validation. By employing an independent dataset distinct from the modeling data for validation, it is possible to transcend the limitations of the model’s original application scope, thereby enhancing its predictive stability in real-world settings. Structurally, one- and two-compartment models are predominantly used to characterize imipenem pharmacokinetics. In particular, the one-compartment model is often preferred in clinical population analyses because it could describe imipenem elimination based on sparse data. However, a three-compartment model was implemented in Li et al. (2020) Study and Ikawa et al. (2008) Study to characterize the PK profile of imipenem.

**TABLE 2:** Results from population pharmacokinetic models of imipenem included in the systematic review.

Study	Author and year	Compartments	Population typical value	Inter-individual variability (IIV)	Residual variability (RV)	Model evaluation method	Software
1	Ikawa et al. (2008)	3-CMT	CL: 9.42 L/h V_1_: 4.66 L V_2_: 5.08 L V_3_: 4.57 L Q_2_: 5.74 L/h Q_3_: 31.6 L/h	CL: 26.9% V_1_: 47.5% V_2_: 37.1% V_3_: 86.2%	Additive: 1.13 mg/L	NR	NONMEM Crystal Ball 2000
2	Lamoth et al. (2009)	1-CMT	CL: 16.2 L/h V: 33.5 L	CL: 17%	Residual error: 59%	NR	NONMEM
3	Yoshizawa et al. (2013)	Neonates 1-CMT Children 2-CMT	Neonates CL_r_: 0.0783 L/h/kg CL_nr_: 0.138 L/h/kg V: 0.466 L/kg Children CL_r_: 0.187 L/h/kg CL_nr_: 0.0711 L/h/kg V_1_: 0.203 L/kg V_2_: 0.0569 L/kg Q: 0.0621 L/h/kg	Neonates CL_r_: 39.2% CL_nr_: 33.4% Children CL_r_: 17.7% CL_nr_: 39.5% V_1_: 17.1%	Neonates Proportional: 25.2% Additive: 0.483 mg/L Children Proportional: 27.9%	GOF parameter sensitivity leverage analyses VPC	NONMEM
4	Couffignal et al. (2014)	2-CMT	CL: 13.2 L/h V_1_: 20.4 L V_2_: 9.8 L Q: 12.2 L/h	CL: 38% V_1_: 31%	Proportional: 33%	GOF VPC NPDE Bootstrap	Monolix
5	Li and Xie (2019)	1-CMT	CL_body_: 6.11 L/h V_d_: 34.2 L	CL_body_: 36.6% V_d_: 47.2%	Proportional: 26.3%	pcVPC NPDE SIR GOF	NONMEM
6	Dong et al. (2019)	2-CMT	CL: 8.6 L/h Q: 0.996 L/h V_1_: 7.2 L V_2_: 6.51 L	CL: 18.8% V_1_: 9.2%	Proportional: 39.5% Additive: 0.205 mg/L	Bootstrap GOF NPDE	NONMEM
7	Li et al. (2020)	3-CMT	CL_c_: 8.825 L/h CL_d_: 0.093 L/h Q_cp_: 1.572 L/h Q_cd_: 0.392 L/h V_c_: 24.264 L V_p_: 33.035 L V_d_: 0.012 L	CL_c_: 35.394% CL_d_: 31.624% Q_cp_: 156.718% V_c_: 54.996% V_p_: 97.659%	Plasma Proportional: 23.655% Additive: 0.584 mg/L Effluent Proportional: 43.652% Additive: 0.384 mg/L	Bootstrap VPC	Phoenix NLME
8	Chen et al. (2020b)	2-CMT	CL: 8.88 L/h Q: 1.74 L/h V_1_: 20.5 L V_2_: 8.86 L	CL: 17.7% V_1_: 14.8%	Proportional: 6.2% Additive: 0.003 mg/L	Bootstrap VPC GOF	NONMEM
9	de Velde et al. (2020)	2-CMT	NONMEM K_e_: 0.637 h^−1^ K_cp_: 0.166 h^−1^ K_pc_: 0.195 h^−1^ V_c_: 29.6 L Pmetrics K_e_: 0.681 h^−1^ K_cp_: 0.374 h^−1^ K_pc_: 0.495 h^−1^ V_c_: 31.1 L	NONMEM K_e_: 19% Pmetrics K_e_: 34% K_cp_: 81.2% K_pc_: 72% V_c_: 42.6%	NONMEM Proportional: 34.8% Pmetrics Gamma: 3.4	VPC NPC GOF Bootstrap	NONMEM and Pmetrics
10	Nguyen et al. (2021)	1-CMT	CL: 7.88 L/h V: 15.1 L	CL: 29.4% V: 10.7%	Proportional: 23.3%	Bootstrap VPC GOF NPDE	Monolix
11	Jaruratanasirikul et al. (2021)	2-CMT	CL: 13.3 L/h V_1_: 13.6 L V_2_: 16.9 L Q: 24.3 L/h	CL: 51% V_1_: 66.9% V_2_: 56%	Proportional: 18.3% Additive: 0.216 mg/L	pcVPC NPDE bootstrap	NONMEM
12	Por et al. (2021)	2-CMT	CL: 15.31 L/h V_1_: 32.67 L V_2_: 41.23 L Q: 11 L/h	CL: 30.5% V_1_: 36.1%	Proportional: 30%	NPDE Bootstrap VPC External validation	Pumas
13	Dao et al. (2022)	1-CMT	CL: 0.21 L/h V: 0.73 L	CL: 20%	Proportional: 37% Additive: 0.04 mg/L	Bootstrap pcVPC GOF	NONMEM
14	Dinh et al. (2022)	2-CMT	CL: 4.79 L/h V_1_: 11.1 h V_2_: 8.82 L Q: 11.1 L/h	CL: 38.7% V_1_: 56.3% V_2_: 69.4% Q: 110%	Proportional: 22.1% Additive: 0.445 mg/L	GOF NPDE VPC Bootstrap	Monolix
15	Bai et al. (2024)	2-CMT	CL: 11.357 L/h Q: 7.645 L/h V_1_: 16.378 L V_2_: 10.904 L	CL: 35.748% V_1_: 89.853% V_2_: 8.319%	Proportional: 30.37%	Bootstrap VPC GOF	Phoenix NLME
16	Lafaurie et al. (2023)	1-CMT	Neutropenia CL: 14.3 L/h V: 20.7 L After neutropenia recovery CL: 10.9 L/h V: 14.5 L	Neutropenia CL: 19.8% V: 17.4% After neutropenia recovery CL: 21.8% V: 27.8%	Neutropenia Proportional: 15.9% Additive: 0.42 mg/L After neutropenia recovery Proportional: 36.1% Additive: 0.3 mg/L	GOF NPDE	Monolix
17	Truong et al. (2025)	2-CMT	CL: 14.6 L/h V_1_: 28.7 L V_2_: 21.4 L Q: 2.9 L/h	CL: 35.9%	Proportional: 38.30%	Bootstrap VPC GOF External validation	NONMEM
18	Wang et al. (2024)	2-CMT	CL: 13.1 L/h V_1_: 11.7 L V_2_: 29.3 L Q: 11.9 L/h	CL: 8.32%	Additive: 0.575 mg/L	GOF pcVPC Bootstrap External validation	NONMEM

CMT, compartment; CL, clearance; CL_c_, clearance of the central compartment; CL_d_, clearance of the dialysis compartment; CL_r_, clearance of plasma drug; CL_nr_, clearance of urine drug; CL_body_, endogenous clearance; Q, intercompartmental clearance; Q_cp_, intercompartment clearance between the central and peripheral compartments; Q_cd_, intercompartment clearance between the central and dialysis compartments; Q_2_, intercompartmental clearance (peripheral compartment 1); Q_3_, intercompartmental clearance (peripheral compartment 2); V_1_, volume of central compartment; V_2_, volume of peripheral compartment/peripheral compartment 1; V_3_, volume of peripheral compartment 2; V_d_, volume of distribution; K_e_, the elimination rate constant; K_cp_, rate constant from the central to peripheral compartment; K_pc_, rate constant from the peripheral to central compartment; GOF, goodness‒of-fit plots; VPC, visual predictive check; NPDE, normalized prediction distribution errors; pcVPC, prediction-corrected visual predictive check; SIR, sampling importance resampling procedure; NR, no record.

**FIGURE 2:**
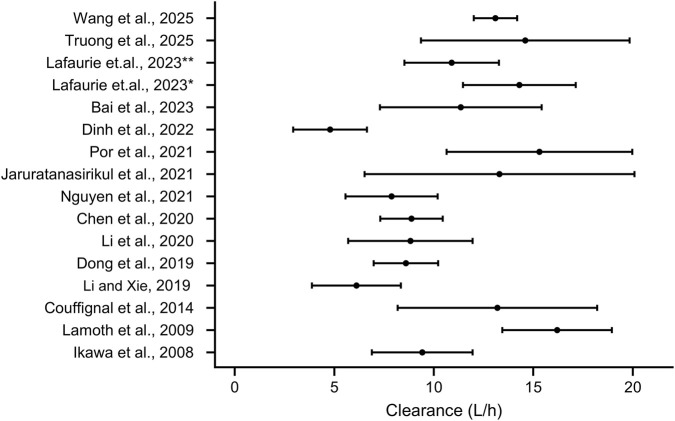
The estimation of imipenem clearance of adults in various study. * indicates patients with neutropenia, ** indicates patients recovery from neutropenia.

The reviewed studies demonstrated considerable variability in the estimated PK parameters of imipenem. The estimations of imipenem Clearance (CL) are shown in Table 2 ad Figure 2, which range from as low as 0.0783 L/h/kg in neonates (Yoshizawa et al., 2013) to as high as 16.2 L/h in febrile neutropenic patients with hematological malignancies (Lamoth et al., 2009). Regarding distribution volumes, in two-compartment models, the central volume of distribution (V_1_) varies between 0.203 L/kg in children (Yoshizawa et al., 2013) and 32.67 L in burn patients undergoing CVVH (Por et al., 2021).

The evaluation of covariates affecting imipenem pharmacokinetics is presented in Table 3. Among the factors assessed, creatinine clearance (CLcr) was the most consistently significant covariate, being identified in 10 out of 11 studies that tested it and serving as the sole predictor in the final models of Nguyen et al. (2021), Bai et al. (2024), Truong et al. (2025), Wang et al. (2024), and Dinh et al. (2022) Study. BW was also frequently retained and was incorporated into 8 out of 14 studies where it was evaluated (Chen et al., 2020b; Couffignal et al., 2014; Dao et al., 2022; Dong et al., 2019; Jaruratanasirikul et al., 2021; Lamoth et al., 2009; Por et al., 2021). In contrast, age was examined in 11 studies but was included in only one final model (Dong et al., 2019), whereas gender was not selected as a significant covariate in any of the 10 studies that considered it. Notable population-specific covariates were also identified. For example, the glomerular filtration rate (GFR) significantly influences imipenem clearance in febrile neutropenic patients with hematological malignancies (de Velde et al., 2020; Jaruratanasirikul et al., 2021; Lamoth et al., 2009). In critically ill patients, imipenem clearance is affected by clinical factors such as burn injury, extracorporeal membrane oxygenation (ECMO), diuresis, the GFR, and the serum ALB concentration. Neonate populations are influenced by BW, gestational age (GA), postnatal age (PNA), and serum creatinine (SCr) (Dao et al., 2022), whereas only CLcr is significant in elderly patients (Wang et al., 2024).

**TABLE 3:** Selection of covariates in the population pharmacokinetic models from the included studies.

Study	Author and year	Covariate analysis method	Covariates screened	Covariates incorporated	Formulation
1	Ikawa et al. (2008)	NR	NR	NR	NR
2	Lamoth et al. (2009)	NR	BW, eGFR_CG_	eGFR_CG_ on CL BW on V	CL = 10.7 + 4.79 × eGFR/100 (mL/min) V = 33.5 × BW/70
3	Yoshizawa et al. (2013)	NR	Age, gender, and dose	NR	NR
4	Couffignal et al. (2014)	Forward inclusion (p < 0.05)	Age, gender, TBW at inclusion and TBW change (between the 4th dose and admission), SAPS II score, the SOFA score, the oedema score, serum ALB, CLcr_4h_, PEEP, arterial P_aO2_F_iO2_ ratio, and the presence of septic shock	CLcr_4h_ on CL TBW and ALB on V_1_	CL = 13.2 × (CLcr/86.4)^0.2^ V_1_ = 20.4 × (BW/77)^1.3^ × (ALB/18)^(−1.1)^
5	Li and Xie, 2019	Forward inclusion (p < 0.05)	Age, gender, BW, patient type (with or without burn), degree of diuresis, urine output, APACHE II score, and %TBSA	Residual diuresis and burn jury on CL	CL_total_ = CL_CRRT_ + CL_body_ CL_CRRT_ = Sd × CRRT_intensity_ × BW/1,000 CL_body_ = 6.11 × (1 + θ_BURN_) × (1 + θ_DIUR_) × e^η_CLbody_^ θ_BURN_ = 0; non-urned patients θ_BURN_ = 0.817; burn patients θ_DIUR_ = 0; patients with anuria θ_DIUR_ = 0.434; patients with oliguria θ_DIUR_ = 0.659; patients with preserved diuresis
6	Dong et al. (2019)	Forward inclusion ΔOFV > 3.84 (p < 0.05) Backward elimination ΔOFV > 6.635 (p < 0.01)	Age, BW, CLcr_Schwartz_	Age, BW, and CLcr_Schwartz_ on CL BW on V_1_, V_2_, and Q	CL = 8.6 × (BW/18)^0.75^ × (age/4.69)^0.265^ × (CLcr/214)^0.509^ V_1_ = 7.2 × (BW/18) V_2_ = 6.51 × (BW/18) Q = 0.996 × (BW/18)^0.75^
7	Li et al. (2020)	Forward inclusion ΔOFV > 6.63 (p < 0.01) Backward elimination ΔOFV > 10.83 (p < 0.001)	Age, gender, weight, sepsis, sepsis shock, ventilatory assistance, course of treatment, AKI, Scr, CLcr_CG_, blood flow, Qd, replacement fluid flow, APACHE II score, and SOFA score	CLcr_CG_ on CL_c_ Qd on CL_d_	CL_c_ = 8.825 × (CLcr/50.896)^0.221^ × e^η_CLc_^ CL_d_ = 0.093 × (Qd/500)^1.944^ × e^η_CLd_^
8	Chen et al., (2020b)	Forward inclusion ΔOFV > 3.84 (p < 0.05) Backward elimination ΔOFV > 7.88 (p < 0.005)	Age, gender, BW, HT, BMI, SCR, CLcr_CG_, ALT, AST, ALB, TBIL, HGB, PLT, ECMO, CRRT, type of infection	CLcr_CG_, BW, and ECMO on CL	With ECMO CL = 8.88 × (CLcr/59.1)^0.295^ × (BW/65.0)^0.306^ × e^1.16^×e^η_CL_^ Without ECMO CL = 8.88 × (CLcr/59.1)^0.295^ × (BW/65.0)^0.306^ × e^η_CL_^
9	de Velde et al. (2020)	Forward inclusion ΔOFV > 3.84 (p < 0.05) Backward elimination ΔOFV > 10.83 (p < 0.001)	TBW, IBW, LBW, eGFR_CG_, eGFR_MDRD-4_, eGFR_CKD-EPI_, and eGFR_Jelliffe_	eGFR_CKD-EPI_ on K_e_	NONMEN K_e_ = 0.637 × ((eGFR_CKD-EPI-abs_)/119)^0.655^ × e^η^ Pmetrics K_e_ = K_ei, med_ × ((eGFR_CKD-EPI-abs_)/119)^K_e(cov)i, med_^
10	Nguyen et al. (2021)	Forward inclusion ΔOFV > 6.635 (p < 0.01) Backward elimination ΔOFV > 10.828 (p < 0.001)	Age, gender, weight, BMI, CLcr_CG_, Anthonisen score, CLcr_MDRD-4_, respiratory distress, and diuretics intake	CLcr_CG_ on CL	CL = 7.88 × (CLcr/75.54)^0.532^ × e^η_CL_^
11	Jaruratanasirikul et al. (2021)	Forward inclusion ΔOFV > 3.84 (p < 0.05) Backward elimination ΔOFV > 6.64 (p < 0.01)	Age, gender, actual BW, ideal BW, ABW, BMI, the use of ECMO support, ECMO type, ECMO flow rate, duration of ECMO, APACHE II scores, SOFA scores, CLcr_CG_, eGFR_MDRD-4_, eGFR_MDRD-6_, eGFR_CKD-EPI_, AKI, mechanical ventilation support, serum ALB, fluid balance, use of inotropes, septic shock, and mean arterial blood pressure	eGFR_CKD-EPI_ on CL ABW on V_1_	CL = 13.3 + 0.112 × (eGFR_CKD-EPI_ -89) V_1_ = 13.8 − 0.348 × (ABW-60)
12	Por et al. (2021)	Forward inclusion ΔOFV > 3.84 (p < 0.05)	Age, total body weight, LBW, CLcr_CG_, TBSA, total second-degree burn surface area, total third-degree burn surface area, serum ALB, urine output, and use of CVVH	BW and ALB on Vc ALB on Vp With CVVH CLcr_CG_, WT on CL Without CVVH BW on CL	Vc = 32.67 × (BW/99.5)^0.74^ × (ALB/2.7)^(−1.17)^ × e^η^Vc Vp = 41.23 × (ALB/2.7)^(−3.68)^ Without CVVH: CL = 15.31 × (CLcr/145.83)^0.46^ × (BW/99.5)^0.33^ × e^ηCL^ With CVVH CL = 13.78 × (BW/99.5)^0.75^ × e^ηCL^ + 1.56
13	Dao et al. (2022)	Forward inclusion Backward elimination	Gender, BW, GA, PNA, PMA, SGA, SCr, and concomitant treatments	BW, GA, PNA, SCr on CL BW on V	CL = 0.21 × (BW/1.16)^0.75^ × (1 + 0.22 × (PNA-21)/21) × (1 + 1.31 × (GA-26.9)/26.9) × (46.6/SCr)^0.2^ V = 0.73 × (BW/1.16)^0.75^
14	Dinh et al. (2022)	COSSAC method	Age, gender, actual BW, serum ALB, CLcr_CG_, UNIT (ICU vs. non-ICU), VASO use and mechanical ventilation	CLcr_CG_ on CL	CL = 4.79 × e^(0.00642 × CLcr)^
15	Bai et al. (2024)	Forward inclusion ΔOFV > 3.84 (p < 0.05) Backward elimination ΔOFV > 6.6 (p < 0.01)	Age, gender, BW, course, Scr, CLcr_CG_, SOFA score, APACHE II score, sepsis, AKI, septic shock and ventilation	CLcr_CG_ on CL	CL = 11.357 × (CLcr/99.896)^0.473^ × e^η^
16	Lafaurie et al. (2023)	NR	NR	NR	NR
17	Truong et al. (2025)	Forward inclusion (p < 0.01) Backward elimination (p < 0.001)	Age, gender, TBW, ABW, IBW, HT, BMI, BSA, UNIT (ICU vs. non-ICU), CLcr_CG_, eGFR_CKD-EPI_, and eGFR_MDRD-4_	CLcr_CG_ on CL	CL = 14.6 × (CLcr_CG_/87.6)^0.462^
18	Wang et al. (2024)	Forward inclusion ΔOFV > 3.84 (p < 0.05) Backward elimination ΔOFV > 6.63 (p < 0.01)	CLcr_CG_, CRP, WBC, and CRRT	CLcr_CG_ on CL	CL = 13.1 × (CLcr/71)^(0.263)^ × e^ηCL^

CL, clearance; CL_c_: clearance of the central compartment; CL_d_, clearance of the dialysis compartment; V_1_, volume of central compartment; Q, intercompartmental clearance; V_2_, Volume of Peripheral Compartment/Peripheral Compartment 1; K_e_, the elimination rate constant; CLcr, clearance creatinine; Qd, dialysate flow; APACHE, acute physiology and chronic health evaluation score; SOFA, sequential organ failure assessment; BMI, body mass index; BW, body weight; HT, height; ALT, alanine transaminase; AST, aspartate transaminase; ALB, albumin; TBIL, total bilirubin in serum; HGB, hemoglobin; PLT, platelet count; ECMO, extracorporeal membrane oxygenation; CRRT, continuous renal replacement therapy; %TBSA, percentage of burned total body surface area; BSA, body surface area; CRRT_intensity_, the sum of ultrafiltration rate and dialysis rate; θ_BURN_, factor for the influence of burn injury on CL_body_; θ_DIUR_ (Oliguria), factor for the influence of oliguria on CL_body_; θ_DIUR_ (Preserved diuresis), factor for the influence of preserved diuresis on CL_body_; CLcr_CG_, clearance creatinine estimated by Cockcroft and Gault equation; CLcr_MDRD-4_, clearance creatinine estimated by four-variable Modification of Diet in Renal Disease equation; AKI, acute kidney injury; Scr, serum creatinine; TBW, total body weight; IBW, ideal body weight; LBW, lean body weight; eGFR_CG_, estimated glomerular filtration rate measured by Cockcroft–Gault equation; eGFR_MDRD-4_, estimated glomerular filtration rate measured by four-variable Modification of Diet in Renal Disease equation; eGFR_CKD-EPI_, estimated glomerular filtration rate measured by the Chronic Kidney Disease Epidemiology Collaboration equation; eGFR_Jelliffe_, estimated glomerular filtration rate measured by the Jelliffe’s equation; CKD-EPI-abs, absolute CKD-EPI (i.e., CKD-EPI, multiplied by BSA); ABW, adjusted body weight; GA, gestational age; PNA, postnatal age; SGA, small for gestational age; PMA, postmenstrual age; SAPS II, score, Simplified Acute Physiology Score; PEEP, positive end-expiratory pressure; P_aO2_F_iO2_, partial pressure of oxygen/fractional inspired oxygen; CLcr_4h_, 4 h creatinine clearance; CRP, C-reactive protein; WBC, white blood cell count; VASO, vasopressor; COSSAC, conditional sampling use for stepwise approach based on correlation tests; NR, no record.

## 4 Discussion

Imipenem is a beta-lactam carbapenem antibiotic characterized by its broad-spectrum antibacterial activity and high stability against beta-lactamases. It is frequently employed as a critical therapeutic agent for severe infections caused by multidrug-resistant pathogens (Armin et al., 2023; Barbier et al., 2023; Kaya et al., 2022). Given its narrow therapeutic window (high doses of imipenem are prone to induce neurotoxicity and nephrotoxicity), pathogens with increased drug resistance and significant interindividual variability in pharmacokinetics, popPK modeling is essential for its optimal use (Chen et al., 2020a; Huo et al., 2020; Martínez Delgado et al., 2022; Slama, 2008). However, although several popPK studies have been published, the sources of PK variability are unclear. Therefore, this review is the first to synthesize recent advances in imipenem popPK and summarize the covariates that significantly affect imipenem exposure.

This systematic review included a total of 18 studies from various countries, five of which were conducted in China. Notably, only three studies enrolled more than 100 patients, whereas the smallest study included only 10 participants. As popPK models rely on group data to estimate parameters, studies with small samples often lack statistical power, making it difficult to accurately identify and quantify the influence of key covariates on imipenem PK. In addition, small sample sizes may significantly constrain the robustness and generalizability of the developed models (Berisha and Liss, 2024). Restricted sample representation can introduce bias, thereby limiting the model’s capacity to reflect the true variability in pharmacokinetic profiles across real-world patient populations, particularly among those with high heterogeneity. Therefore, larger-scale studies are warranted in the future to increase the reliability of individualized dosing strategies for imipenem.

Among the studies included in this review, NONMEM was the most extensively utilized tool for popPK analysis. The two-compartment model is most frequently employed to characterize the PK of imipenem, which closely aligns with its *in vivo* distribution properties. Following administration, imipenem rapidly distributes into highly perfused tissues and moderately perfused organs, a phase described by the intercompartment clearance (Q) between the central (V_1_) and peripheral (V_2_) compartments. This is followed by a comparatively slower elimination phase, governed predominantly by the total CL. The two-compartment model effectively captures the characteristic feature of the drug concentration‒time curve, which exhibits an initial rapid decline (distribution phase) followed by a slower decline (elimination phase), striking an optimal balance between model complexity and biological plausibility.

Although the two-compartment model is most commonly applied, some investigations have explored the use of one- or three-compartment models. The one-compartment model benefits from structural simplicity and fewer parameters, facilitating easier fitting and convergence. However, its major limitation lies in the inability to accurately depict the pronounced distribution phase of imipenem, often resulting in underestimation of early plasma concentrations and potential bias in AUC estimation. In contrast, a three-compartment model could theoretically offer a more refined characterization of the slow distribution phase into deep tissues, such as adipose or poorly perfused regions, potentially enabling a more precise depiction of the *in vivo* processes (van de Bool et al., 2015). This advantage becomes particularly relevant in studies investigating imipenem PK in nonplasma compartments, for example, peritoneal fluid (Ikawa et al., 2008) and the dialysate of CRRT (Li et al., 2020). By employing a three-compartment model, researchers are better able to quantify drug disposition in these specific biological compartments, which often exhibit kinetics distinct from both central and shallow peripheral compartments. Nevertheless, this model demands extensively rich sampling data for reliable identification of all the parameters. Given the typically sparse nature of clinical data, the three-compartment model often exhibits unstable convergence, high uncertainty in parameter estimates, and a tendency for overfitting (Rambiritch et al., 2016).

Substantial interindividual variability in PK parameters was observed across studies utilizing a two-compartment model (CL: 4.79–15.31 L/h; V_1_: 7.2–32.67 L; V_2_: 2.9–41.23 L; Q: 0.996–24.3 L/h). Some studies focused on the same clinical group, but the intra-group variability in PK parameters was also large. For example, the CL in adult critical ill patients varied from 4.79 to 14.6 L/h. This heterogeneity can be attributed primarily to the considerable pathophysiological diversity among the investigated patient populations, which included critically ill subjects with or without ECMO support, elderly patients, children with hematological malignancies, burn patients with or without receiving CVVH, and critically ill patients with suspected ventilator-associated pneumonia. These groups differ markedly in terms of fluid balance, organ function, and hemodynamic status. Notably, the highest CL value (15.31 L/h) in two-compartment model was reported in burn patients with or without CVVH (Por et al., 2021). This elevation is explained by the hyperdynamic circulatory state characteristic of major burns, which involves increased cardiac output and enhanced renal blood flow, thereby accelerating the elimination of imipenem from the kidney (Stanojcic et al., 2018). Furthermore, CVVH contributes to drug elimination by providing an additional clearance pathway (Corona et al., 2022). Similarly, markedly enlarged volumes are frequently documented in critically ill patients, particularly those with sepsis or severe burns (Dickinson and Kollef, 2011; Pruskowski, 2020; Roberts and Lipman, 2009). This phenomenon is associated with capillary leakage, tissue edema, and expansion of the extracellular fluid volume (Roberts and Lipman, 2009). ECMO support may also further increase the apparent distribution volume due to drug adsorption to the circuit (Ahsman et al., 2010; Jelliffe, 2016). It is important to identify and successfully incorporate covariate that can explain the inter-individual variability in the final model.

The majority of studies included in this review predominantly involved critically ill patients. This focus can be attributed to the complex clinical presentations, rapid disease progression, and high mortality rates observed in this population, which consequently make them a priority for clinical intervention and research (Reignier et al., 2025). However, other patient groups also warrant considerable investigation, particularly elderly, infant, and child patients. Elderly patients often present with multiple comorbidities, declining physiological function, and altered PK profiles, predisposing them to adverse outcomes and complicated clinical courses (Medellín-Garibay et al., 2022). Neo et al. reported that carbapenems induced seizures in 2.4% of elderly patients, a prevalence substantially higher than the 0.2%–0.7% range documented in the literature (Neo et al., 2020). In contrast, neonates and children are characterized by ongoing growth and development, resulting in significant differences from adults in terms of organ function, immune status, and drug response. These distinctions lead to unique disease manifestations, therapeutic requirements, and prognostic features. In support of this, a study by Pevzner et al. demonstrated that imipenem/cilastatin was associated with more pronounced nephrotoxicity in neonates, underscoring the need for greater caution in antibiotic selection and dosing in this vulnerable group (Pevzner et al., 2023). Despite the urgent clinical needs in these specific populations, only a limited number of popPK studies targeting these populations were identified in the present review. This indicates a significant research gap, highlighting the necessity for future investigations to prioritize the pharmacokinetics of imipenem in these distinct patient subgroups.

Renal function is known to be the primary determinant of imipenem clearance (Gorham et al., 2022). Consequently, popPK analyses are needed to quantify the impact of renal impairment and other covariates in populations with varying degrees of kidney function. Renal function can be estimated by different equations, commonly CLcr by Cockcroft-Gault equation, eGFR by MDRD and CKD-EPI equations. However, these equations differ in accuracy, especially for special populations like the critically ill, obese and elderly. Among the studies included in this review, CLcr was identified as a statistically significant covariate in ten investigations, eGFR in three, and Scr in one. This distribution reflects the prevalent utilization of CLcr as a routine biomarker for renal function assessment and dosage individualization in clinical practice. When renal function decreases and glomerular filtration capacity becomes impaired, Scr, a waste product of muscle metabolism, cannot be effectively eliminated. Consequently, elevated Scr levels directly indicate diminished renal filtration function. However, Scr is considered a relatively delayed indicator because of the considerable functional reserve of the kidneys; a significant reduction in the GFR must occur before Scr increases noticeably (Romano et al., 2013; Yoo et al., 2019). Furthermore, Scr levels are influenced by factors such as age, sex, muscle mass, and diet (Levey et al., 1999). Therefore, Scr is commonly employed in conjunction with age, sex, and ethnicity for estimating the GFR or CLcr via established equations to better reflect renal functional status in clinical settings (Brown et al., 2013; Inker et al., 2021; Levey et al., 2006; Winter et al., 2012). Recently, Mitton et al. reported that the GFR estimated via the CKD-EPI equation is not related to the plasma level of imipenem in critically ill patients (Mitton et al., 2022). This indicated that the accuracy in estimating renal function of different formulations may introduce bias in covariate effect estimation, and further influence the performance of final model and accuracy of dosing recommendation. It suggests that the plasma concentration of imipenem in critically ill patients cannot be predicted solely on the basis of GFR and that therapeutic drug monitoring (TDM) is a safe and effective approach to ensure precision dosing. Additionally, future studies should include more samples and covariates to facilitate the development of a more precise imipenem popPK model.

In the study by Li et al., conventional renal biomarkers such as CLcr, GFR, and Scr were not identified as statistically significant covariates (Li and Xie, 2019). This observation is explained by the fact that their research focused on critically ill patients undergoing CRRT. In this population, drug clearance is substantially influenced by extracorporeal support, thereby limiting the ability of traditional renal function indicators to accurately reflect the actual elimination rate of medications. Alternatively, the model developed by Li et al. (2020) incorporates residual diuresis as a meaningful variable. The clinical relevance of utilizing residual diuresis for dose adjustment has been supported by previous PK investigations. For example, Yu et al. introduced residual diuresis as a significant covariate when developing a popPK model for vancomycin in critically ill patients receiving CRRT (Yu et al., 2023). Similarly, Ulldemolins et al. demonstrated its considerable impact on optimizing meropenem dosing regimens in a comparable patient population (Ulldemolins et al., 2015). Collectively, these findings underscore that residual diuresis serves as an essential and nonnegligible parameter for guiding individualized drug therapy in patients receiving CRRT.

BW was identified as another key covariate characterizing imipenem distribution and elimination and was incorporated into the final model in six of the reviewed studies. For hydrophilic antibacterial agents such as imipenem, PK behavior is closely associated with extracellular fluid volume and renal function, and BW serves as a fundamental metric for estimating body composition and normalizing renal function (Alobaid et al., 2016; Meng et al., 2017). This parameter is particularly relevant in pediatric populations or patients with significant weight fluctuations, where it forms the basis for individualized dosing regimens (Gade et al., 2018; Natale et al., 2017). Consequently, it is frequently included in pharmacokinetic models to increase its predictive accuracy.

In addition to BW and renal function markers, age is commonly considered a demographic covariate, although its significance varies across populations (Sanghavi et al., 2024). This review revealed that age is a meaningful covariate in models involving children (Dong et al., 2019) and neonates (Dao et al., 2022). During early development, age serves as a surrogate for dynamic changes in body size, body composition, and renal function. Keij et al. provided age-specific dose recommendations for pooled popPK studies of intravenous and oral amoxicillin in neonates (Keij et al., 2023). In contrast, the influence of age is often supplanted by more direct physiological or biochemical indicators. Therefore, most adult popPK models do not retain age as a significant covariate.

Notably, most of the included studies relied solely on internal validation, such as bootstrapping, GOF plots, and VPC, whereas only three investigations performed external validation (Por et al., 2021; Truong et al., 2025; Wang et al., 2024). If only internal data were used for validation, the good predictive performance of the model would only be reflected in its own center, making its generalizability to other patient groups uncertain. Therefore, future research should prioritize rigorous external evaluation of these models through multicenter data to verify their predictive ability across diverse clinical settings.

## 5 Conclusion

In conclusion, of the 18 studies systematically evaluated in this review, popPK models for imipenem have been successfully established across different patient subpopulations. A two-compartment model was predominantly employed to characterize the imipenem popPK model. Notably, markers of renal function were consistently identified as the most significant covariates influencing imipenem exposure, which aligns with imipenem’s primary renal elimination. BW and patient age were also demonstrated to substantially impact PK parameters, necessitating their consideration during therapy. Future research should focus on quantifying the effects of under investigated covariates. Importantly, rigorous external validation was conducted to verify the predictive robustness and general applicability of these models in diverse clinical environments.

## Data Availability

The original contributions presented in the study are included in the article/Supplementary Material, further inquiries can be directed to the corresponding authors.
