# Supplementary material for: Population pharmacokinetics of imipenem in different populations for individualized dosing: a systematic review
Source: Front Pharmacol. 2026 Jan 14;16:1738055. doi: 10.3389/fphar.2025.1738055 (PMC12847419; doi:10.3389/fphar.2025.1738055)
Supplement: Supplementary file 1 [file Supplementaryfile1.zip › Table S1.docx]

Table S1. Overview of the included clinical study protocols in the systematic review.

| **Study** | **Author and year** | **Dosage** | **Sampling time point** | **Assay** |
| --- | --- | --- | --- | --- |
| 1 | Ikawa et al., 2008 | 500 mg | 0.5, 1, 2, 3, 4, 5, and 6 h after administration | HPLC-UV |
| 2 | Lamoth et al., 2009 | 500 mg (q6h) | 10 min before and at a median of 2 h (range, 0.5 to 4 h) after the start of the infusion | HPLC-UV |
| 3 | Yoshizawa et al., 2013 | Neonate: 10.0-37.3 mg/kg  Child: 8.70-30.0 mg/kg | NR | NR |
| 4 | Couffignal et al., 2014 | 500-1000 mg (q8h) | 0, 0.5, 1, 2, 5 and 8 h after the fourth infusion | HPLC-UV |
| 5 | Li et al., 2018 | 500 mg (q6h, q8h)  750 mg (q6h)  1000 mg (q6h, q8h) | NR | NR |
| 6 | Dong et al., 2019 | 15-25 mg/kg (q6h) | 3 to 5 min, 0.5 to 1 h after the end of infusion, and 2 to 6 h after the start of infusion | HPLC-UV |
| 7 | Li et al., 2020 | 500-1000 mg | 0.5, 1, 1.5, 2, 3, 4, 6, and 8 h after imipenem administration | HPLC-UV |
| 8 | Chen et al., 2020 | 250 mg (q12h)  500 mg (q6h, q8h, q12h)  500 mg (qm) and 250mg (qn)  1000 mg (q6h, q8h, q12h) | TDM data: 3 h and 0.5 h before the next administration;  Blood samples with ECMO: before administration and 0.5, 1, 2, 3, 6, and 8 h after the beginning of infusion after the 4th dose with ECMO and after its withdrawal | LC-MS/MS |
| 9 | Velde et al., 2020 | 500 mg (q6h) | Peak, intermediate, trough concentration points | HPLC-UV |
| 10 | Nguyen et al., 2021 | 500 mg (q6h, q8h, q12h)  1000 mg (q8h, q12h) | 0.5 h after infusion of the third dose and 1-2 h prior to the 4th dose | HPLC-UV |
| 11 | Jaruratanasirikul et al., 2021 | 250-500 mg (q6-12h)  500 mg (q6h)  1000 mg (q6h, q8h) | 0, 0-0.5, 0.5-2, 2-4, and 4-12 h after administration | HPLC-UV |
| 12 | Por et al., 2021 | 250 mg, 500 mg,  or 1000 mg (q6h) | 0 (trough), 0.5-8 h after administration | HPLC-UV |
| 13 | Dao et al., 2022 | 15-20 mg/kg (q8-12h) | C_max_ (1-2 h after infusion start), at C_min_ (under steady-state conditions, before the fourth dose in general) or both | LC-MS/MS |
| 14 | Dinh et al., 2022 | 500 (q6h, q8h)  1000 mg (q8h) | 0, 0.25, 0.67, 1.5, and 7 h after administration | HPLC-UV |
| 15 | Bai et al., 2023 | 500 mg (q6h, q8h);  1000 mg (q8h) | 0.5, 1, 1.5, 2, 3, 4, 6, and 8 h before and after imipenem administration | HPLC-UV |
| 16 | Lafaurie et.al, 2023 | 1000 mg (q8h)  500 mg (q6h) | Immediately before, and at 0.5, 1, 2, 3, 4, 6 and 8 h after administration | HPLC-UV |
| 17 | Truong et al., 2025 | NR | Peak, intermediate, trough concentration points | HPLC-UV |
| 18 | Wang et al., 2025 | 250-1000 mg (q6-12h) | Trough concentration points | HPLC-UV |

LC‒MS/MS, liquid chromatography‒tandem mass spectrometry; HPLC‒UV, high-performance liquid chromatography‒ultraviolet detection; HPLC, high-performance liquid chromatography; h, hour; NR, no record.
